# Supplementary material for: Stable nebulization and muco‐trapping properties of regdanvimab/IN‐006 support its development as a potent, dose‐saving inhaled therapy for COVID‐19
Source: Bioeng Transl Med. 2022 Aug 30;8(1):e10391. doi: 10.1002/btm2.10391 (PMC9537933; doi:10.1002/btm2.10391)
Supplement: Supplementary file 1 — Appendix S1 Supporting Information [file BTM2-8-e10391-s001.docx]

| **Age** | **Sex** | **Smoker or Exposure to smoker** | **Race/Ethnicity** |
| --- | --- | --- | --- |
| 60 | F | N | Caucasian |
| 13 | F | N | Caucasian |
| 65 | M | N | Caucasian |
| 56 | M | Y | Hispanic |
| 54 | F | N | Caucasian |
| 31 | M | N | African American |
| 21 | M | N | Caucasian |
| 64 | F | N | Caucasian |
| 55 | M | N | Hispanic |
| 64 | F | N | African American |

**Supplementary Table 1. Characteristics of Airway Mucus Donors**. Characteristics of the donors that provided fresh AM samples that were used in the *in vitro* virus trapping studies described in Figure 1.

| **No.** | **Sample** | **Number of particles**  **2 - 10 μm (per mL)** | **Comparison of pre- and post-neb particle counts** |
| --- | --- | --- | --- |
| 1 | Pre-nebulization #1 | 677 | p=.103 |
| 2 | Pre-nebulization #2 | 882 |  |
| 3 | Pre-nebulization #3 | 1480 |  |
| 4 | Post-nebulization #1 | 1713 |  |
| 5 | Post-nebulization #2 | 3458 |  |
| 6 | Post-nebulization #3 | 2420 |  |

**Supplementary Table 2. Impact of nebulization on particulate matter.** High accuracy (HIAC) liquid particle counts for pre- and post-nebulized formulations of mAb. Notably, particulates in this size range for a topical product are less problematic than for IV-administered products, as humans are exposed to micron-scale particles on a regular basis. Number of particles in pre- and post-nebulized samples were compared via paired, two-tailed T-test.

**
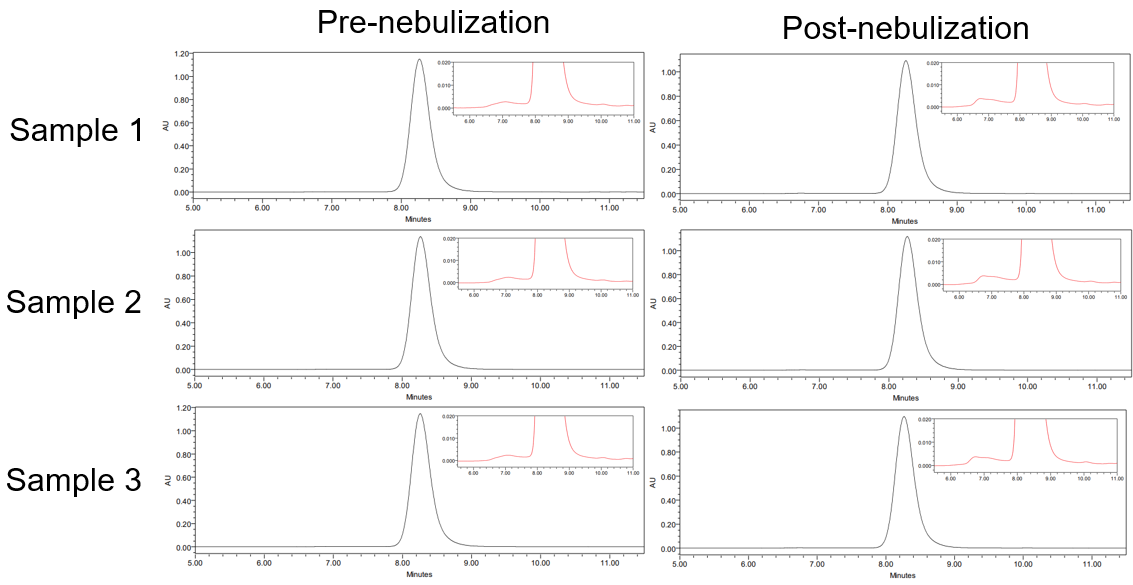
**

**Supplementary Figure 1. Size Exclusion Chromatography.** Comparing three each of pre- and post-nebulized samples of IN-006, there was no apparent difference in mAb population on size exclusion chromatography. Inset drawings in red represent an enlarged view of the base of the curve shown in black in the same panel.

**
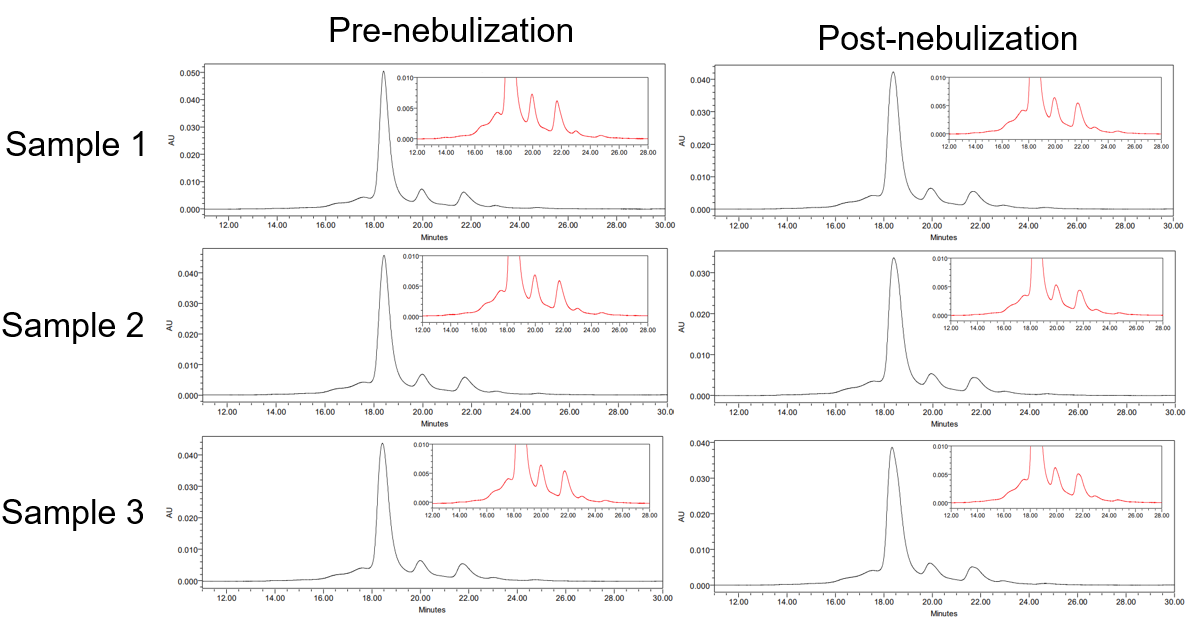
**

**Supplementary Figure 2. Ion Exchange Chromatography pre- and post-nebulization.** Comparing three each of pre- and post-nebulized samples of IN-006, there was no apparent difference in mAb population on ion exclusion chromatography. Inset drawings in red represent an enlarged view of the base of the curve shown in black in the same panel.

**
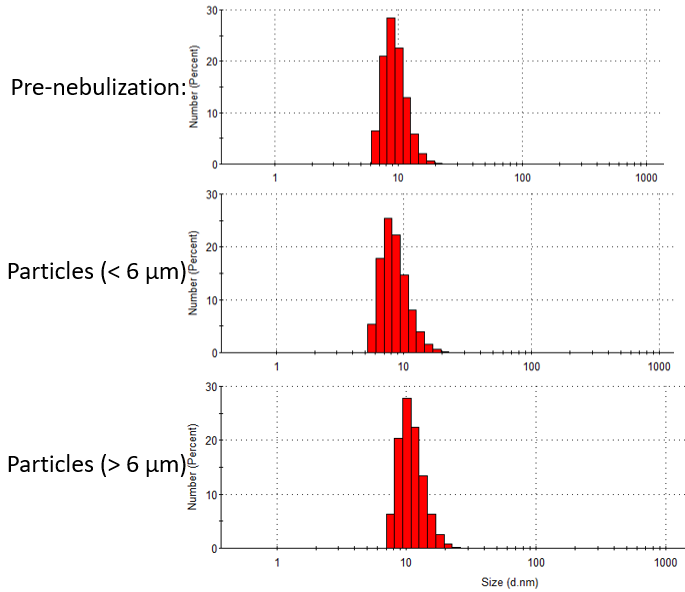
**

**Supplementary Figure 3. Dynamic Light Scattering pre- and post-nebulization.** IN-006 samples were analyzed via dynamic light scattering (number weighted) to assess for the presence of aggregates. The three conditions shown are pre-nebulized material and material collected into the upper (> 6 µm aerosol) or lower (< 6 µm) chamber of a glass impinger following nebulization. There was no apparent increase in aggregated species of IN-006 in either the larger or smaller aerosols collected.

**Supplementary Video 1:** SARS-CoV-2 VLPs in fresh human AM without addition of IN-006

**Supplementary Video 2:** SARS-CoV-2 VLPs in fresh human AM with addition of IN-006, demonstrating trapping
